# People with Visual Impairment Continue to Experience Difficulties in Their Daily Lives that Affect Their Health-related Quality of Life after the COVID-19 Pandemic

**DOI:** 10.31662/jmaj.2023-0120

**Published:** 2023-11-27

**Authors:** Yoshiyasu Ito, Hana Kiyohara, Kenji Awamura, Chizuru Yamaoka

**Affiliations:** 1 College of Nursing Art and Science, University of Hyogo, Akashi, Japan; 2 Department of Nursing, Graduate School of Health Sciences, Kobe University, Kobe, Japan

**Keywords:** postpandemic, difficulty in daily life, health-related quality of life, visual impairment

The coronavirus disease 2019 (COVID-19) pandemic has caused many difficulties in people’s lives worldwide. This particularly applies to people with visual impairment (VI), who have difficulties visually recognizing objects, including recognizing objects tactilely and performing daily activities, such as moving. However, when people were forced to take countermeasures against infection during the pandemic, contact with objects and people was limited. Thus, during this period, people with VI experienced many difficulties in daily life ^(1), (2), (3)^, and their health was negatively affected ^(4)^.

Recently, the number of COVID-19 cases has declined worldwide, and on May 5, 2023, the World Health Organization chief declared that COVID-19 was no longer a global health emergency ^(5)^. In January 2023, the Japanese government decided to categorize COVID-19 as category V of infectious disease, a decision that went into effect on May 8, 2023 ^(6), (7)^. Nonetheless, these decisions from authoritative organizations neither mean that infection control measures are no longer necessary nor guarantee that there will be no future outbreaks of COVID-19. However, many citizens have begun to return to their prepandemic routines, starting with being more economically active. In particular, lowering the infectious disease category encouraged people to resume the lives they had before the spread of infection. Indeed, the number of new cases in Japan has declined to less than 10,000 since March 2023, with 6,806 cases reported as of March 31, 2023 ^(8)^. In a survey conducted at approximately the same time, 86.2% of respondents answered that they planned to go out as much or as actively as they did before the spread of infection ^(9)^.

However, in this transition to the postpandemic period, it is unclear whether the daily lives of people with VI and their health, which have been associated with many difficulties, are improving. Therefore, we conducted this study to determine the current status of difficulties in daily life among people with VI and its association with health-related quality of life (HRQOL) and life satisfaction after the pandemic.

This study was a cross-sectional online survey conducted via Microsoft Forms (Microsoft Corporation, Redmond, WA, USA). The data for this study were collected from April 1 2023 to June 1, 2023. The participants were recruited through snowball sampling. During recruitment, we initially recruited three participants belonging to different organizations of people with VI located in Hyogo Prefecture, Japan. These participants used the mailing lists of their organizations to recruit participants via e-mail. Participants who were not on the mailing lists were asked to participate in the study through individual e-mails from other participants who belonged to the same organization. Participants were asked to participate by receiving the study’s description via e-mail containing a URL to participate and respond to the online survey. Consent for participation and publication of the study’s information was obtained from all participants via an online form. This study was approved by the Institutional Review Board of the College of Nursing Art and Science, Hyogo University (Approval number: 2022F05).

We used the online survey to collect participants’ sociodemographic characteristics, difficulties in daily life, HRQOL, and life satisfaction. We developed and used an original 32-item questionnaire to evaluate participants’ experiences of difficulties in daily life after the pandemic. The questionnaire was developed based on the results of a preliminary qualitative study, which conducted semistructured interviews of eight people with VI to elicit the nature of their difficulties in daily life during the COVID-19 pandemic. Thirty-two items with content validity were selected during discussions among our research teams to be used as the questions in this study ^(10)^. For each question item, the participants responded with a “Yes” or “No,” indicating whether they had experienced the described situation.

HRQOL was measured using the ED-5D-5L ^(11)^, a scale for assessing the HRQOL Index ranging from 0.025 to 1.000, with high scores indicating high HRQOL. Scores are converted from response patterns to five items (degree of mobility, self-care, usual activities, pain/discomfort, and anxiety/depression). In this study, we used the Japanese version of the ED-5D-5L ^(12)^. Following previous studies, life satisfaction was measured using an 11-point Likert scale ranging from “extremely unsatisfied” (0 points) to “extremely satisfied” (10 points) ^(13)^.

Regarding statistical analysis, to examine the associations between experiences of difficulties in daily life and HRQOL and life satisfaction among people with VI after the pandemic, the Mann-Whitney U test was used to compare the differences in median HRQOL and life satisfaction between the groups that answered “Yes” and “No” on each of the 32 items to assess participants’ experiences of difficulties in daily life.

A total of 65 participants completed the online survey. The participants’ characteristics are listed in Table 1. The most common experience of difficulties in daily life for participants was “*It is difficult to communicate through partitions, ventilation, and masks used for infection control because it is difficult to hear the other party’s voice*” (84.6%), followed by “*It is difficult to cope with social distancing*” (81.5%). These were not associated with HRQOL or life satisfaction. Experiences of difficulties in daily life associated with HRQOL that participants reported frequently were “*There are fewer* *opportunities to partake in hobbies and leisure activities*” (72.3%), “*There are now fewer opportunities to engage with supporters and non-visually impaired people*” (69.2%), and “*It has become difficult to ask for assistance from others in the city*” (66.2%). Life satisfaction was associated with “*I feel that my relationship with family and friends has improved*” (32.3%), “*I am not able to play the role I want in my job*” (40.4%), and “*I don’t feel like starting a new job*” (32.7%). All associations between the experiences of difficulties in daily life, HRQOL, and life satisfaction among people with VI after the pandemic are reported in Table 2.

**Table 1.** Demographic Characteristics of Participants.

Characteristics		n (%)
Sex	Male	42 (64.6)
	Female	22 (33.8)
	Other	1 (1.5)
Age, year	Mean ± SD	66.0 ± 11.4
Grade of Visual Impairment^ a^	Grade 1	44 (67.7)
	Grade 2	19 (29.2)
	Grade 3	1 (1.5)
	Grade 6	1 (1.5)
Living with family	Yes	47 (72.3)
	No	18 (27.7)
Can go out alone	Yes	52 (80.0)
	No	13 (20.0)
Guide helper service user^ b^	Yes	44 (67.7)
	No	21 (32.3)
Employment status	Employed	44 (67.7)
	Unemployed	21 (32.3)
ED-5D-5L, HRQOL	Mean ± SD	0.8 ± 0.2
	Median (IQR)	0.8 (0.7-1.0)
Life satisfaction	Mean ± SD	6.1 ± 2.3
	Median (IQR)	7.0 (5.0-8.0)

Abbreviations: HRQOL, Health-Related Quality of Life; IQR, Interquartile Range ^a.^ Legal visual impairment grades in Japan are established and classified following the Japanese Physical Disability Welfare Law. There are six grades based on the sum of visual function and corrected visual acuity of both eyes, with low grades indicating severe visual impairment. ^b.^ Guide helper in Japan refers to a person who assists people with visual impairment in their daily activities, provides them with information, and helps them read/write.

**Table 2.** Associations between Experiences of Difficulties in Daily Life, and HRQOL and Life Satisfaction among the People with Visual Impairment after the COVID-19 Pandemic.

Questionnaire items and response on experiences of difficulties in daily life		n (%)	ED-5D-6L, HRQOL		Life Satisfaction	
			Median (IQR)	P-value^a^	Median (IQR)	P-value^a^
*It is difficult to communicate through partitions, ventilation, and masks used for infection control because it is difficult to hear the other party’s voice*	*“Yes”*	55 (84.6)	0.82 (0.71-0.89)	.065	7.00 (5.00-8.00)	.346
	*“No”*	10(15.4)	0.94 (0.83-1.00)		5.50 (4.00-7.00)	
*From the perspective of infection control, I feel it is difficult to touch and check things when shopping or traveling*	*“Yes”*	52(80.0)	0.82 (0.72-0.89)	.518	7.00 (5.00-8.00)	.473
	*“No”*	13(20.0)	0.87 (0.75-1.00)		6.00 (5.00-7.00)	
*I don’t know what kind of infection control measures (alcohol disinfection, temperature measurement, etc.) are required on the go*	*“Yes”*	44(67.7)	0.82 (0.73-0.95)	.519	6.00 (5.00-7.00)	.204
	*“No”*	21(32.3)	0.89 (0.71-1.00)		8.00 (5.00-8.00)	
*It is difficult to cope with social distancing*	*“Yes”*	53(81.5)	0.82 (0.71-0.89)	.162	6.00 (5.00-8.00)	.419
	*“No”*	12(18.5)	0.88 (0.81-1.00)		7.00 (5.00-8.00)	
*Feeling anxious about infection when contacting guide helpers* ^b^	*“Yes”*	10(22.7)	0.81 (0.71-0.89)	.923	6.00 (5.00-8.00)	.649
	*“No”*	34(77.3)	0.81 (0.71-0.89)		6.50 (5.00-8.00)	
*Unless I know the guide helper well (familiar/previously involved), I feel anxious about infection* ^b^	*“Yes”*	13(29.5)	0.75 (0.66-0.89)	.224	5.00 (4.00-8.00)	.317
	*“No”*	31(70.5)	0.82 (0.73-0.89)		7.00 (5.00-8.00)	
*Concerns about infectious diseases made it difficult to use guide helpers* ^b^	*“Yes”*	15 (34.1)	**0.74 (0.70-0.79)**	**.032**	6.00 (4.50-7.50)	.27
	*“No”*	29(65.9)	**0.89 (0.74-1.00)**		7.00 (5.00-8.00)	
*I began to feel apologetic for using guide helpers* ^b^	*“Yes”*	20(45.5)	**0.74 (0.59-0.86)**	**.032**	5.00 (5.00-8.00)	.311
	*“No”*	24(54.5)	**0.88 (0.76-1.00)**		7.00 (5.00-8.00)	
*It has become difficult to ask for assistance from others in the city*	*“Yes”*	43 (66.2)	**0.80 (0.70-0.89)**	**.009**	6.00 (5.00-8.00)	.394
	*“No”*	22 (33.8)	**0.95 (0.80-1.00)**		7.00 (5.00-8.00)	
*There were fewer opportunities to receive support from strangers*	*“Yes”*	36(55.4)	**0.78 (0.70-0.89)**	**.013**	6.00 (5.00-8.00)	.178
	*“No”*	29(44.6)	**0.89 (0.82-1.00)**		7.00 (5.00-8.00)	
*There are now fewer opportunities to engage with supporters and non-visually impaired people*	*“Yes”*	45(69.2)	**0.80 (0.70-0.89)**	**.005**	6.00 (5.00-8.00)	.191
	*“No”*	20(30.8)	**1.00 (0.81-1.00)**		7.00 (5.50-8.00)	
*Even if I ask for help, such as guidance, I feel that they are being wary*	*“Yes”*	29(44.6)	**0.80 (0.71-0.89)**	**.019**	6.00 (4.00-8.00)	.344
	*“No”*	36(55.4)	**0.89 (0.74-1.00)**		7.00 (5.00-8.00)	
*Enthusiasm for hobbies and leisure activities decreased*	*“Yes”*	31(47.7)	**0.78 (0.72-0.85)**	**.019**	6.00 (5.00-7.00)	.435
	*“No”*	34(52.3)	**0.89 (0.75-1.00)**		7.00 (5.00-8.00)	
*There are fewer opportunities to partake in hobbies and leisure activities*	*“Yes”*	47(72.3)	**0.80 (0.70-0.89)**	**.003**	6.00 (5.00-8.00)	.367
	*“No”*	18(27.7)	**1.00 (0.83-1.00)**		7.00 (5.00-8.00)	
*I am not able to do the hobbies and leisure activities I want*	*“Yes”*	48(73.8)	0.82 (0.70-0.89)	.1	6.00 (5.00-8.00)	.143
	*“No”*	17(26.2)	0.89 (0.76-1.00)		7.00 (5.00-8.00)	
*I don’t feel like starting a new hobby or leisure activity*	*“Yes”*	28(43.1)	0.79 (0.72-0.89)	.124	6.00 (5.00-8.00)	.676
	*“No”*	37(56.9)	0.87 (0.75-1.00)		7.00 (5.00-8.00)	
*Instead of using guide helpers, I have asked my family and friends more and more*	*“Yes”*	18(27.7)	0.82 (0.74-0.89)	.728	6.00 (5.00-8.00)	.911
	*“No”*	47(72.3)	0.83 (0.71-1.00)		7.00 (5.00-8.00)	
*I spend more time with my family and friends*	*“Yes”*	27(41.5)	0.87 (0.74-0.95)	.554	7.00 (5.00-8.00)	.32
	*“No”*	38(58.5)	0.82 (0.71-1.00)		6.00 (5.00-8.00)	
*I feel that my relationship with family and friends has improved*	*“Yes”*	21(32.3)	0.89 (0.82-0.89)	.057	**8.00 (5.00-8.00)**	**.025**
	*“No”*	44(67.7)	0.79 (0.69-1.00)		**6.00 (5.00-7.00)**
*I care more about my family and friends*	*“Yes”*	49(75.4)	0.82 (0.73-0.89)	.258	7.00 (5.00-8.00)	.653
	*“No”*	16(24.6)	0.88 (0.71-1.00)		6.50 (4.50-7.50)
*Opportunities for communication with others using ICT have increased*	*“Yes”*	52(80.0)	0.82 (0.72-0.89)	.471	7.00 (5.00-8.00)	.109
	*“No”*	13(20.0)	0.89 (0.73-1.00)		5.00 (4.00-6.00)
*The use of ICT has increased opportunities for visually impaired people to communicate with each other*	*“Yes”*	45(69.2)	0.83 (0.71-1.00)	.763	7.00 (5.00-8.00)	.525
	*“No”*	20(30.8)	0.80 (0.74-0.95)		6.00 (5.00-7.50)
*I do not use ICT because it is difficult to operate*	*“Yes”*	18 (27.7)	0.81 (0.70-0.89)	.304	6.50 (5.00-7.00)	.721
	*“No”*	47 (72.3)	0.87 (0.73-1.00)		7.00 (5.00-8.00)
*I would like support in using ICT*	*“Yes”*	45 (69.2)	**0.80 (0.66-0.89)**	**.009**	7.00 (5.00-8.00)	.628
	*“No”*	20 (30.8)	**0.89 (0.80-1.00)**		6.50 (5.00-8.00)
*I have fewer opportunities to go out due to the COVID-19 pandemic*	*“Yes”*	48(73.8)	0.82 (0.72-0.89)	.157	6.50 (5.00-8.00)	.844
	*“No”*	17(26.2)	0.89 (0.75-1.00)		7.00 (5.00-8.00)
*When I go out, I am more patient about where I want to go (places other than unnecessary and nonurgent, eating out, etc.) because I think it will cause trouble for others*	*“Yes”*	40(61.5)	0.81 (0.71-0.89)	.073	7.00 (5.00-8.00)	.995
	*“No”*	25(38.5)	0.89 (0.75-1.00)		6.00 (5.00-8.00)
*I often refrain from going out because I am worried about the eyes (eyes) around me*	*“Yes”*	21(32.3)	0.80 (0.71-0.89)	.357	7.00 (5.00-8.00)	.966
	*“No”*	44(67.7)	0.85 (0.73-1.00)		6.50 (5.00-8.00)	
*By using home delivery and mail order, we have reduced the opportunities to make contact with people as much as possible*	*“Yes”*	32(49.2)	0.79 (0.72-0.89)	.263	6.00 (5.00-8.00)	.262
	*“No”*	33(50.8)	0.89 (0.73-1.00)		7.00 (5.00-8.00)	
*I feel less motivated to work^ c^*	*“Yes”*	18(34.6)	**0.75 (0.71-0.82)**	**.002**	5.50 (4.00-7.00)	.087
	*“No”*	34(65.4)	**0.89 (0.80-1.00)**		7.00 (5.00-8.00)	
*The amount of work has decreased^ c^*	*“Yes”*	29(55.8)	0.82 (0.74-0.89)	.502	6.00 (5.00-8.00)	.373
	*“No”*	23(44.2)	0.87 (0.75-1.00)		7.00 (5.00-8.00)	
*I am not able to play the role I want in my job^ c^*	*“Yes”*	21(40.4)	**0.74 (0.62-0.82)**	**<.000**	**6.00 (5.00-7.00)**	**.025**
	*“No”*	31(59.6)	**0.89 (0.81-1.00)**		**7.00 (5.00-8.00)**	
*I don’t feel like starting a new job^ c^*	*“Yes”*	17(32.7)	**0.75 (0.71-0.82)**	**.010**	**5.00 (4.00-7.00)**	**.021**
	*“No”*	35(67.3)	**0.89 (0.79-1.00)**		**7.00 (5.00-8.00)**	

Abbreviations: HRQOL, Health-Related Quality of Life; ICT, Information and Communication Technology; IQR, Interquartile Range ^a^ Mann-Whitney U-test ^b^ 21 participants did not use guide helpers ^c^ 13 participants were unemployed

This study’s results highlight that many people with VI continued to have few opportunities to engage with supporters and others after the pandemic, making it difficult for them to seek help, thereby limiting their HRQOL. During the pandemic, people with VI were not well supported and were dissatisfied with the support they received from health and social services ^(14)^. This study found that even after the pandemic, over 60% of participants experienced few opportunities to engage with supporters and others, thereby indicating that people with VI may continue to face difficulties in obtaining support―as was the case during the pandemic. Notably, many people with VI reported difficulty asking others for assistance. This is important as previous studies have shown that perceptions of social support influence formal and informal help-seeking behaviors ^(15)^. This study’s findings suggest that support for people with VI, which has been reduced owing to the pandemic, has resulted in their perception of low social support, resulting in reduced help-seeking behavior. Therefore, to improve the HRQOL of people with VI in the postpandemic period, strategies are needed to raise awareness regarding social support, such as providing specific information on the service resources currently available to people with VI, including formal and informal services that have resumed after the pandemic.

This study has several limitations. First, this cross-sectional study was conducted after the COVID-19 pandemic, and no data were collected before or during the pandemic; therefore, it was impossible to compare the current situation to prior conditions. Second, this study included a small number of participants. Hence, the analysis could not be adjusted for participants’ background, such as age, employment status, etc. Third, the response rate for this study is unknown because the snowball sampling method was used to recruit participants within their institutions, along with recruitment via e-mail. This may have biased the sample toward those with access to electronic devices and those with mild VI levels, thereby compromising representativeness. Finally, the original 32-item questionnaire used to assess participants’ experiences of difficulties in daily living after the pandemic has not been assessed for validity and reliability.

However, to our knowledge, this study is the first to determine the experience of difficulties in daily life among people with VI and its association with HRQOL and life satisfaction after the pandemic. This study’s results provide valuable data to support people with VI after the pandemic and indicate the need for further research.

## Article Information

### Conflicts of Interest

None

### Sources of Funding

This work was supported by JR-West Relief Foundation.

### Acknowledgement

We thank Editage (www.editage.jp) for English-language editing.

### Author Contributions

Yoshiyasu Ito: Conceptualization, Methodology, Formal analysis, Writing-original draft, Investigation; Hana Kiyohara: Conceptualization, Methodology, Formal analysis, Writing-review & editing; Kenji Awamura: Conceptualization, Methodology, Investigation, Resources, Writing - review & editing; Chizuru Yamaoka: Conceptualization, Methodology, Supervision, Funding acquisition, Supervision, Project administration.

### Approval by Institutional Review Board (IRB)

This study was approved by the Institutional Review Board of the Research Ethics Committee, College of Nursing Art & Science and Research Institute of Nursing Care for People and Community, University of Hyogo, Japan (approval no.: 2022F05, approval date: 13 January 2023).
